# Impact of Social Media Use on HIV Testing and Related Mediator among Men Who Have Sex with Men in Shandong Province, China

**DOI:** 10.3390/ijerph20075383

**Published:** 2023-04-04

**Authors:** Daisy Aurore Steinely Mboussi, Yuxi Lin, Lovel Fornah, Wei Ma

**Affiliations:** Department of Epidemiology, School of Public Health, Cheeloo College of Medicine, Shandong University, Jinan 250012, China

**Keywords:** HIV testing, men who have sex with men, social media, HIV facility-based testing, HIV self-testing

## Abstract

In China, HIV testing is the important first step of HIV prevention and treatment cascades but is not widely adopted by men who have sex with men (MSM). However, social media has been increasingly used to promote and improve HIV testing. This study aimed to assess the impact of social media use on HIV testing and explore the mediator in the relationship between social media and HIV testing. The authors conducted a cross-sectional study among 935 MSM participants in 11 cities in Shandong Province from 14 September to 6 December 2020. Multivariable regression was conducted to assess the relationship between social media use and HIV testing uptake, and mediation analysis was used to assess the mediation effect of HIV testing self-efficacy in the relationship between social media use and HIV testing. The present study findings showed that social media use was related to HIV testing (aOR = 3.024, 95% CI: 1.869, 4.892) and HIV self-testing uptake (aOR = 1.894, 95% CI: 1.228, 2.922), but was not related to HIV facility-based testing (aOR = 1.709, 95% CI: 0.806, 3.625, *p* = 0.162). A mediation effect of HIV testing self-efficacy was found between social media use and HIV testing (indirect effect: 0.13, 95% CI: 0.01, 0.29), facility-based testing (indirect effect: 0.05, 95% CI: 0.003, 0.16), and self-testing uptake (indirect effect: 0.07, 95% CI: 0.01, 0.16). Social media could be an effective tool to improve HIV testing, and healthcare providers should pay more attention to HIV testing self-efficacy in HIV testing promotion.

## 1. Introduction

The human immunodeficiency virus (HIV) epidemic is a global issue that affects all races, tribes, and social economic backgrounds. In 2020, there were roughly 1.5 million new HIV infections worldwide [1]. IDU (injection drug use) and MSM (men who have sex with men) seem to be the groups most affected by the HIV epidemic. However, in terms of the raw number of HIV cases, the MSM epidemic is by far the most severe. MSM represent a significant group for HIV infection and transmission, particularly in China, where the MSM had the fastest-rising HIV infection rates over the previous ten years [2]. In China, MSM accounted for 23.0% of new reported HIV infection cases in October 2019, which increased from 14.7% of new HIV cases in 2011 [3]. Due to multiple partners and high-risk sexual activities, MSM are 25 times more likely to be infected with HIV than the general population [4]. According to data from China’s HIV surveillance system, the prevalence of HIV among MSM has increased by about 8% over the last five years, and MSM have become a crucial population for HIV prevention and control [5].

HIV testing is anticipated to be a crucial step in HIV prevention and treatment cascade. The Joint United Nations Program on HIV/AIDS endorsed the 90-90-90 target in 2014 for bringing the HIV epidemic under worldwide control. The first 90 target, representing 90% of all persons living with HIV who know their HIV status, is the most difficult to meet, even in high-income countries. To reach this target, it is essential to increase HIV testing uptake and frequency among key populations such as MSM [6]. MSM should be tested for HIV at least once a year, according to the Centers for Disease Control and Prevention (CDC) recommendations, but ideally, every three to six months if they have had a new sexual partner since their last test [7]. However, only 47% of MSM in China have willingly visited HIV testing facilities [8,9]. HIV testing uptake is still low [10], and effective strategies should be taken to improve HIV testing uptake.

Furthermore, considering that many MSM use social networking platforms to find sex partners worldwide [11], healthcare practitioners increasingly rely on social media to create health promotion materials, disseminate information, and create peer-mentored education programs [12]. In addition, a Taiwanese study discovered that popular internet opinion leaders could help promote HIV testing among MSM through social media networks [13]. Similarly, England created an internet-based social marketing effort to encourage MSM to be tested for HIV [14]. Furthermore, previous studies in China also conducted programs to improve HIV testing uptake using social media among MSM [15,16,17]. However, a previous study highlighted concerns about the impact of social media use on HIV testing among MSM, who may be less likely to frequently test for HIV while also engaging in more unprotected behaviors [18].

Given that there are conflicting findings regarding the relationship between using social media and HIV testing, and that few studies in China have examined the potential mediator in this relationship, this study surveyed MSM in 11 cities in Shandong Province, China, to determine the effect of social media use on HIV testing and to investigate the mediator in this relationship.

## 2. Materials and Methods

### 2.1. Participants and Recruitment

A cross-sectional study was conducted from 14 September to 6 December 2020, among MSM in 11 cities of Shandong Province, China. The eligibility criteria to participate were: (1) be 18 years old or older, (2) live in the study city, (3) have not been tested for HIV in the past three months, (4) not be living with HIV or have never been tested for HIV, and (5) be willing to provide informed consent.

Participants were recruited online through Blued, a banner advertisement considered the biggest gay dating application in China, and recruited by the staff of community-based organizations (CBOs) in each study city. Eligible participants were encouraged to invite no more than 5 MSM from their social network. An online questionnaire was developed using software called Sojump, and all participants were emailed a link to the questionnaire via WeChat (a popular social contact application). Participants were informed of the study’s terms and conditions, including privacy and confidentiality. All participants who filled out the questionnaire could get an allowance of 8 USD.

### 2.2. Measurements

The socio-demographic characteristics of participants were assessed, including age, time of living in the study city, marital status, monthly income, educational degree, sexual orientation, disclosure of sexual orientation to others (including family members, friends, sexual partners or other people except for doctors), and disclosure of sexual orientation to doctors. Behavioral variables consisted of condomless sex and a self-reported history of HIV testing (including both facility-based and self-testing). Likert scales were used to evaluate psychological traits such as HIV testing self-efficacy, social norms around HIV testing, and stigma associated with HIV. Participants were also questioned regarding the purpose of seeking information online and the platforms they usually used.

### 2.3. Statistical Analysis

Descriptive analysis was performed to assess the participant characteristics, the specific purpose of searching for information about HIV testing online, and the platforms used. To identify the covariates when evaluating the relationships between social media use and HIV testing uptake, including HIV facility-based testing and self-testing, the correlations were examined between demographic characteristics, sexual behaviors, social media use, and HIV testing uptake in the participants’ lifetimes. The variables related to social media use and HIV testing were included as covariates in logistic regression.

To assess the mediation effect of HIV testing self-efficacy in the relationship between social media use and HIV testing, we conducted a mediation analysis using PROCESS in SPSS 26.0. The estimate of the mediated effect and 95% confidence interval (CI) were reported. The mediated effect was defined as statistically significant when the 95% CI did not contain zero. For stratified analysis, we evaluated the effect of social media use on different types of HIV testing, including facility-based testing and self-testing. A two-sided significance level of alpha = 0.05 was used for all analyses in SPSS software, version 26.0.

## 3. Results

### 3.1. Participants and Social Media Use

A total of 935 participants were enrolled in this study. Most participants were younger than 30 years old (64.3%), never married (72.8%), and had a college degree or higher (67.3%). Most participants ever searched for information on HIV testing through the Internet (89.2%) and tested for HIV (84.0) in their lifetime (Table 1).

The main purpose of searching through the Internet was to search the information and knowledge about HIV testing (68.3%), followed by searching the locations of HIV testing facilities and discussing HIV testing with others (Table 2). The common platforms participants used to search for information were Baidu and Sogou. Moreover, they used social media, particularly MSM-focused websites, the Centers for Disease Control and Prevention’s official website, application websites, and Q&A websites (Table 3).

### 3.2. Relationship between Social Media Use and HIV Testing Uptake

**Figure 1:**
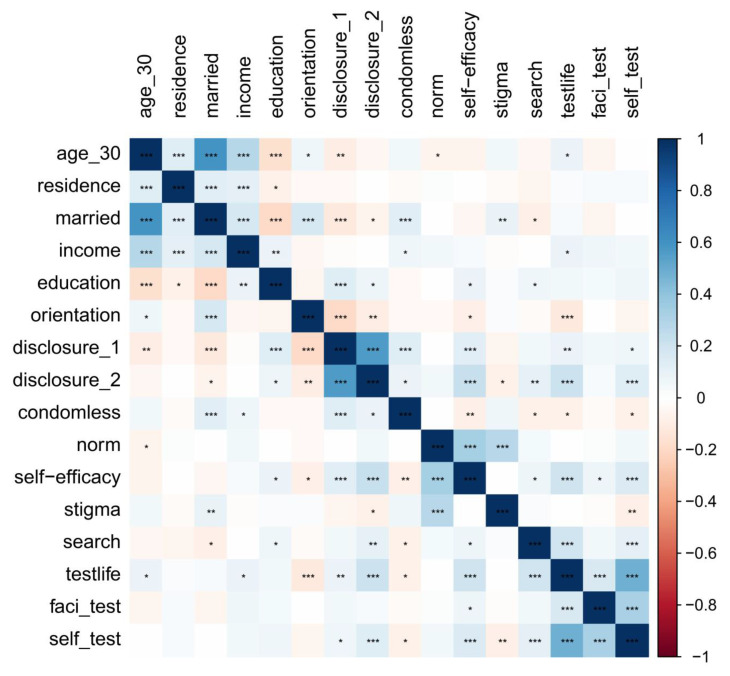
Correlation between demographic characteristics, sexual behaviours, social media use, and HIV testing uptake. Note. ***, *p* < 0.001; **, *p* < 0.01; *, *p* < 0.05.

Social media use was found to be correlated with marital status, educational level, reported sexual behavior with men to doctors, condom-free sexual behavior, and HIV testing self-efficacy (*p* < 0.05). HIV testing uptake was associated with age, income, sexual orientation, disclosed sexual behaviors with men to others and doctors, condomless sexual behaviors, HIV testing self-efficacy, and social media use (*p* < 0.05). Thus, disclosed sexual behaviors with men to doctors, condomless sexual behaviors, and HIV testing self-efficacy were identified as covariates when exploring the effect of social media use on HIV testing uptake (Figure 1).

Table 4 shows the results of the multivariate regression analysis after adjusting the covariates. Participants who searched for information on HIV testing through the Internet were more likely to test for HIV (aOR = 3.024, 95% CI: 1.869, 4.892, *p* < 0.001). In stratified analysis across the type of HIV testing, searching for information on HIV testing through the Internet was related to HIV self-testing uptake (aOR = 1.894, 95% CI: 1.228, 2.922, *p* = 0.004) but was not related to HIV facility-based testing (aOR = 1.709, 95% CI: 0.806, 3.625, *p* = 0.162).

### 3.3. The Mediator in the Relationship between Social Media Use and HIV Testing Uptake

**Figure 2:**
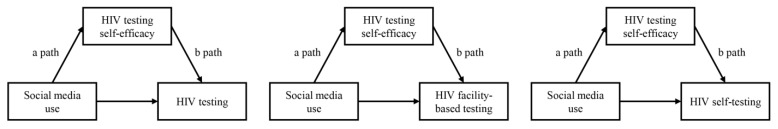
The path of the mediated effect of HIV testing self-efficacy in the relationship between social media use and HIV testing.

Figure 2 shows the path of the mediated effect of HIV testing self-efficacy in the relationship between social media use and HIV testing (including HIV facility-based testing and self-testing), only HIV facility-based testing, and only HIV self-testing. Table 5 presents the estimates and 95% CIs for the “a” path, “b” path, and the mediated effects of HIV testing self-efficacy in these relationships. In the relationship between social media use and HIV testing uptake, the mediated effect of HIV testing self-efficacy was statistically significant (indirect effect: 0.13, 95% CI: 0.01,0.29). The mediated effect of HIV testing self-efficacy was also statistically significant in the relationships between social media use and HIV self-testing (indirect effect: 0.05, 95% CI: 0.003,0.16), and between social media use and HIV facility-based testing uptake (indirect effect: 0.07, 95% CI: 0.01,0.16).

## 4. Discussion

This cross-sectional study surveyed MSM in 11 cities in Shandong Province, China, to assess the impact of social media use on HIV testing and explore the mediator in the relationship between social media and HIV testing. In summary, social media use positively impacts HIV testing, and HIV testing self-efficacy has a mediated effect on this relationship. This research provides insights on HIV testing promotion through social media among MSM.

This result joins the previous studies conducted in different cities in China, which also found an association between social media use and HIV testing uptake, and found that social media acts as a useful tool to promote HIV testing [17,19]. A nationwide cross-sectional survey in China showed that social media use, particularly on multifunctional platforms and with contributing behaviors, is correlated with HIV testing among MSM [17]. Another cluster randomized controlled trial demonstrated that interventions delivered through social media could increase the HIV testing uptake among MSM in China [19]. Social media was demonstrated to be a useful tool in increasing knowledge about HIV testing and improving the perception of the risk of HIV infection, and then promoting the uptake of HIV testing among MSM. Compared with traditional methods, social media, and dating applications could reach more MSM due to their convenience and privacy protection. Therefore, social media should be used more for health promotion. These results are also applicable to other high-risk populations. In addition, this present study also indicated that the main purpose of searching using social media was to search for information and knowledge about HIV testing, and the main platforms used were Baidu and Sougou, inconsistent with a previous study, which demonstrated that social media could be used to push pop-up advertisements about HIV testing [20]. Given that a previous study also indicated that internet technologies are becoming more prevalent and that they might increase sexual risk behaviors, particularly among high-risk groups [21], it was suggested that healthcare providers should publish more authoritative knowledge on HIV testing, and at the same time, the intensive supervision of the knowledge on HIV testing online should be strengthened, especially on popular search engines among MSM. In order to better meet the needs of MSM for information about HIV testing, official healthcare institutions could provide HIV testing counselling services through social media platforms, such as an official website or WeChat official account, which makes it possible for MSM to obtain information without disclosing their sexual orientation or privacy.

Few studies have explored the mediator in the relationship between social media use and HIV testing. This study found that HIV testing self-efficacy was a mediator in the relationship between social media use and HIV testing (HIV self-testing and HIV facility-based testing), which implied that HIV testing self-efficacy might be a crucial factor in increasing HIV testing. HIV testing self-efficacy refers to a person’s expectation of being able to conduct HIV testing [22]. According to Bandura’s theory, the higher the individual’s self-efficacy, the higher the possibility of completing a certain behavior [23]. Previous studies also pointed out that HIV testing self-efficacy is an important psychological factor in promoting HIV testing [24]. These findings provide a guide for healthcare providers in HIV testing promotion using social media. Information to improve HIV testing self-efficacy should be given more attention on social media, including publishing others’ experiences with HIV testing, a peer-leader call for having a test on social networks, and publishing instructions for an HIV self-testing kit.

This study also found an association between disclosed orientation to the doctors and HIV testing. Referrals for MSM who disclosed their sexual orientation to their healthcare provider might get more recommendations for HIV testing than those who did not disclose. A previous survey conducted in China also indicated that men who disclosed to healthcare professionals were more likely to test for HIV compared to men who disclosed to family members [25]. In this study, 64.8% of participants disclosed their orientation to others, and 37.0% did not disclose it to doctors. A systematic review evaluated that the disclosure rates varied across subgroups and study settings, ranging from 16% to 90%, with a median of 61% [26]. It was suggested that the disclosure rate to doctors should be improved in China. Effective strategies should be implemented to encourage MSM to disclose same-sex behaviors and meet their specific medical needs, including providing appropriate training for healthcare providers and creating gay-friendly clinical settings [26].

This study is subject to several limitations. First, due to the cross-sectional design, this study can indicate the correlation but can not test the causality relationship. Second, most participants were recruited online using Blued and by the staff of CBOs. These conclusions therefore cannot be extended to MSM who do not utilize Blued or contact CBOs. Third, using the self-reported HIV testing status can lead to information bias. However, the anonymous questionnaire could reduce this bias to some extent.

## 5. Conclusions

Social media could be an effective tool to improve HIV testing, and healthcare providers should pay more attention to HIV testing self-efficacy in HIV testing promotion. Our findings will help plan the promotion of HIV testing and improve the HIV testing rate.

## Figures and Tables

**Table 1:** Characteristics of participants.

Characteristics	Category	*n* (%)
Age median (IQR), year		26.0 (23.0–32.0)
Age group, year	<30	601 (64.3)
	≥30	334 (35.7)
Time of living in the cities	≤2 years	79 (8.4)
	>2 years	856 (91.6)
Marital status	Never married	681 (72.8)
	Married	172 (18.4)
	Divorced/widowed	82 (8.8)
Monthly income, USD	<250	143 (15.3)
	251–500	139 (14.9)
	501–800	441 (47.2)
	801–1250	161 (17.2)
	>1250	51 (5.4)
Educational degree	High school or lower	306 (32.7)
	College or higher	629 (67.3)
Sexual orientation	Homosexuality	668 (71.4)
	Other	267 (28.6)
Disclosure of sexual orientation to others	Not disclosed to others	329 (35.2)
	Disclosed to others	606 (64.8)
Disclosure of sexual orientation to doctors	Not disclosed to a doctor	589 (63.0)
	Disclosed to doctor	346 (37.0)
Condomless sex	No	621 (66.4)
	Yes	314 (33.6)
HIV testing social norm (Mean ± SD)		17.41 ± 2.39
HIV testing self-efficacy (Mean ± SD)		19.07 ± 2.89
HIV-related stigma (Mean ± SD)		21.09 ± 4.38
Search information on HIV testing through the web	No	101 (10.8)
	Yes	834 (89.2)
Ever tested for HIV	No	150 (16.0)
	Yes	785 (84.0)
HIV facility-based testing	No	816 (87.3)
	Yes	119 (12.7)
HIV self-testing	No	415 (44.4)
	Yes	520 (55.6)

**Table 2:** The purpose of searching through the Internet.

Purpose	N (%)
To search the information about HIV testing	639 (68.3)
To search locations of HIV testing facilities	308 (32.9)
To search the HIV testing process	240 (25.7)
To discuss HIV testing with others	128 (13.7)

**Table 3:** The platforms used to search for information about HIV testing.

Platforms	N (%)
Search engines (such as Baidu and Sogou)	674 (72.1)
Social media (e.g., WeChat, Microblog, QQ)	207 (22.1)
Special websites with HIV testing information (such as Guangtong, Danlan)	184 (19.7)
Official websites of the Centers for Disease Control and Prevention	178 (19.0)
Application (e.g., Blued, Jackd, Grindr, Aloha)	169 (18.1)
Q&A websites on all topics (such as Baidu Knows, Aiwen, Zhihu)	133 (14.2)

**Table 4:** Factors related to HIV testing in their lifetime among MSM.

Characteristics	Category	HIV Testing		HIV Facility-Based Testing		HIV Self-Testing	
		aOR (95% CI)	*p*	aOR (95% CI)	*p*	aOR (95% CI)	*p*
Disclosure of sexual orientation	Not disclosed to a doctor	Ref.				Ref.	
	Disclosed to doctor	3.764 (2.271, 6.241)	<0.001			1.593 (1.198, 2.119)	0.001
Condomless sex	No	Ref.				Ref.	
	Yes	0.697 (0.474, 1.027)	0.068			0.739 (0.557, 0.980)	0.036
Search information on HIV testing through the web	No	Ref.		Ref.		Ref.	
	Yes	3.024 (1.869, 4.892)	<0.001	1.709 (0.806, 3.625)	0.162	1.894 (1.228, 2.922)	0.004
HIV testing self-efficacy		1.168 (1.092, 1.251)	<0.001	1.078 (1.007, 1.155)	0.031	1.088 (1.037, 1.141)	0.001

**Table 5:** Mediation effect of HIV self-efficacy between the relationship between social media use and HIV testing.

Social Media Use	a Path		b Path		Mediated Effect	
	Est (se)	*p*	Est (se)	*p*	Est (se)	95% CI
HIV testing						
No	Ref.		Ref.			
Yes	0.68 (0.30)	0.025	0.19 (0.03)	<0.001	0.13 (0.07)	[0.01, 0.29]
HIV facility-based testing						
No	Ref.		Ref.			
Yes	0.68 (0.30)	0.025	0.08 (0.04)	0.031	0.05 (0.04)	[0.003, 0.16]
HIV self-testing						
No	Ref.		Ref.			
Yes	0.68 (0.30)	0.025	0.10 (0.02)	<0.001	0.07 (0.04)	[0.01, 0.16]

## Data Availability

All data was included in the manuscript. You can contact the corresponding author for more information by writing.
